# When Communities Pull Their Weight: The Economic Costs of an Integrated Agriculture and Nutrition Home-Grown Preschool Meal Intervention in Malawi

**DOI:** 10.1177/0379572120986693

**Published:** 2021-04-21

**Authors:** Amy Margolies, Aulo Gelli, Roshan Daryanani, Aisha Twalibu, Carol Levin

**Affiliations:** 1 8357 International Food Policy Research Institute, Washington, DC, USA; 2 Department of Global Health, 7284 University of Washington, Seattle, WA, USA

**Keywords:** costs, cost-efficiency, school meals, integrated agriculture and nutrition, nutrition-sensitive, early childhood development

## Abstract

**Background:**

Community-based preschool meals can provide an effective platform for implementing integrated agriculture and nutrition programs. However, there is little evidence on the costs and cost-efficiency of implementing these types of multisectoral interventions.

**Objectives:**

Assess the economic costs and cost-efficiency of implementing an effective integrated nutrition-sensitive intervention through a preschool platform in Malawi, including community-level contributions.

**Methods:**

The Strengthening Economic Evaluation for Multisectoral Strategies for Nutrition (SEEMS-Nutrition) framework and methods were applied to assess financial and economic costs of the intervention. A mixed-methods approach was used to measure and allocate costs for program activities and inputs using financial expenditure data combined with micro-costing. All costs were allocated to input and expenditure categories using the SEEMS-Nutrition framework. To facilitate comparisons with existing school meals programs, activities were also mapped against a standardized school feeding supply chain framework.

**Results:**

The total annualized cost of the program was US$197 377, inclusive of both financial and economic costs. The annual economic cost of the program ranged from US$160 per preschool child to US$41 per beneficiary. The principal drivers of cost by program activity were training (46%), school meals provision (19%), monitoring and evaluation (12%), and establishing and running community groups (6.5%). Notably, community contributions accounted for 25% and were driven by food donations and volunteer labor.

**Conclusions:**

Cost per beneficiary estimates of implementing an integrated agriculture–nutrition intervention through an early childhood development platform compare favorably with similar interventions. Further research is needed that applies a standardized economic evaluation framework to such multisectoral interventions.

## Background

The scaling up of nutrition-specific interventions will not be sufficient to meet global targets for improving nutrition.^1^ Contributions from other sectors are required to meet these goals. Agriculture has strong potential due to the manifold pathways through which it can influence nutrition.^2^ A recently updated review of the contributions of agricultural programs in improving nutrition suggested that these types of programs are particularly effective at increasing intake of nutritious foods and improving diet quality when they include strong behavior change communication (BCC) and women’s empowerment interventions.^3^ However, the same review highlighted that despite progress in documenting agriculture and nutrition linkages, there are important gaps in the evidence on the costs of implementation.

School feeding and early childhood development (ECD) programs are two potential platforms recommended for delivering nutrition interventions to young children.^4^ One justification for using ECD platforms to deliver nutrition interventions is the potential for synergies between nutrition and child development.^5^ Another justification is that with the priority for nutrition interventions on the first 1000 days, preschool children (2-6 years old) are not covered by nutrition interventions until they start school. Though preschoolers may have less potential compared to younger children to benefit from nutrition interventions in terms of growth, they also face important nutritional needs, including consuming a nutritious and healthy diet that meets their nutrient requirements. In Malawi, 37% of children are affected by stunting with 4% of under-5 children having acute malnutrition.^6^ Although there is a lack of data or evidence on the intrahousehold distribution of food, it is reported that populations in most African countries consume diets that are nutritionally inadequate for child development.^7,8^

Integrating ECD and nutrition services provides a bridge in nutrition programming among children beyond the first 2 years. However, although there are data on the costs of stand-alone ECD,^9^ the evidence on costs and benefits of multisectoral interventions that combine nutrition and ECD is not well established.

This is particularly relevant as school feeding programs operate in nearly every country in the world and the scale-up of school feeding has been a key response to economic crises of the past decade.^10^ Though school feeding interventions are not always designed with a primary objective of improving nutrition, program design can include features that enhance the potential for nutrition outcomes, including optimizing meal planning and using school meals as a platform for nutrition-related behavior change.^11^ A model known as Home-Grown School Feeding (HGSF), involving linking the provision of goods and services for school feeding to smallholder farmers and the community, has received renewed attention in terms of its potential to improve nutrition- and agriculture-related outcomes.^4^ However, the review of the evidence of school feeding jointly undertaken by the World Bank and World Food Program highlighted that “more accurate estimates of costs are an important area for future research.”^12^ Where cost estimates of school feeding programs exist, they generally do not capture contributions made by the community at the school level. Only one study in the literature includes full costing across the program supply chain.^13^ This study analyzed on-site meal programs in 4 countries in sub-Saharan Africa, namely, Kenya, Lesotho, Malawi, and the Gambia. School-level contributions to school feeding ranged from 0% in Lesotho to 15% in Kenya. The gap in the evidence on the trade-offs in terms of the costs and cost-efficiency of different school feeding models is an important limitation for program and policy stakeholders in selecting effective school feeding models within budgetary constraints.^12^

This study aims to (1) address the evidence gap on costs and cost-efficiency by evaluating the total incremental costs of implementing an integrated nutrition and agriculture intervention through a community-based preschool meal platform in Malawi and (2) present the incremental costs of implementing the integrated intervention through the application of a methodological approach designed for multisectoral nutrition strategies. A comprehensive cost analysis captured all program inputs, including contributions from the government, implementing agency, and the communities served by the intervention. Ascertaining economic costs allows for the identification of the main cost drivers and opportunities for cost containment. In particular, this study examines the “hidden” costs of such programs, such as the opportunity cost of the time invested in the program by parents and caregivers involved in the preschool meal service delivery.

### Program and Context

In Malawi, preschools known as community-based childcare centers (CBCCs) are one of the main components of Malawi’s national Early Childhood Health and Development (ECD) program (the other component being parenting groups). There are an estimated 11 000 CBCCs in Malawi, which aim to provide “safe, stimulating environments, access to health and nutrition services, and training for parents and caregivers.”^14^ Given that the lack of regular food provision at CBCCs causes absenteeism and even CBCC closure, in 2015 Save the Children introduced an integrated nutrition and agriculture package into the existing ECD program. This program, or the Nutrition Embedding Evaluation Program Impact Evaluation (NEEPIE) program, aimed to help communities to improve the consistency and nutritional quality of food produced and provided at CBCCs as well as to enhance the quality of food at the household level.

### Salient Features of the Integrated Agriculture and Nutrition Intervention

**Figure 1.**
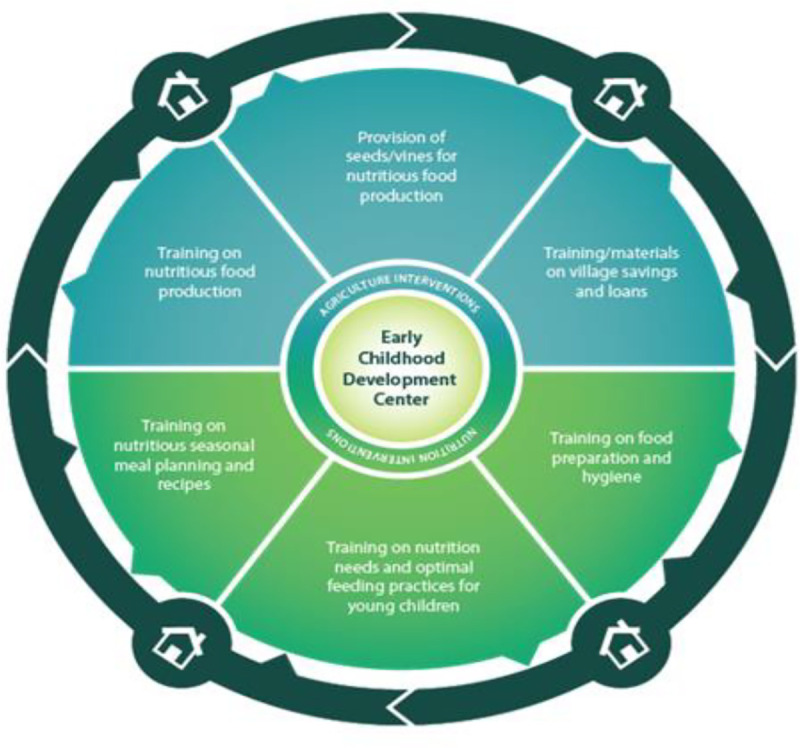
Program design (Nutrition Embedded Evaluation Program Impact Evaluation).

Save the Children and Chancellor College (University of Malawi) developed a CBCC-based nutrition and agriculture intervention drawing on existing nutrition, agriculture, and livelihood materials, using CBCC gardens and school meals as a platform for training and practicing new agricultural, meal preparation, and planning techniques to be reproduced at home. The control group were communities whose preschools were supported with the basic package of Save the Children’s ECD program while the treatment group communities also received this basic ECD package with the additional activities of the integrated agriculture–nutrition intervention. The key aspects of the program were training and provision of inputs. Trainings covered agricultural topics, nutrition, as well as how to manage VSL groups. The components of the program are outlined in Figure 1.

### The Agriculture Intervention

Parents, CBCC management committee representatives, lead farmers and community agents were trained over 3 days before the 2 main planting seasons. The training focused on land preparation, selection of nutritious crops, agriculture production techniques, pest and disease management, manure making and application, harvesting, storage and processing, and chicken rearing. Participants were trained by government agriculture extension development officers (AEDOs), and the CBCC garden was used as a demonstration site for new agricultural techniques. The AEDOs visited the community once a month to check on progress and address problems. Households and CBCCs also received crop and vegetable seeds, such as amaranth and carrot, as well as sweet potato vines and poultry chicks. Village savings and loans (VSL) groups were formed to help households save and access funds to start small businesses and purchase supplies for the CBCC and for use in emergencies.

### The Nutrition Intervention

The CBCC management committee members, CBCC caregivers, lead farmers, and parents received a 3-day nutrition training to help them plan and prepare nutritious meals for the CBCCs and for their households. The training focused on essential nutrition and hygiene practices, seasonal nutritious food selection, food storage, preservation and preparation, CBCC meal planning, and menu adaptation for the household and younger children. The AEDOs and nutrition assistants directed trainings, leading cooking demonstrations and small group practice sessions. The program was designed to have parents rotate preparation of CBCC meals. Thus, parents continued practicing recipes at the CBCC, which were then replicated at home.

Inputs were provided as seeds for nutritious foods for household production and for CBCCs for consumption by preschool children and their families.

**Figure 2.**
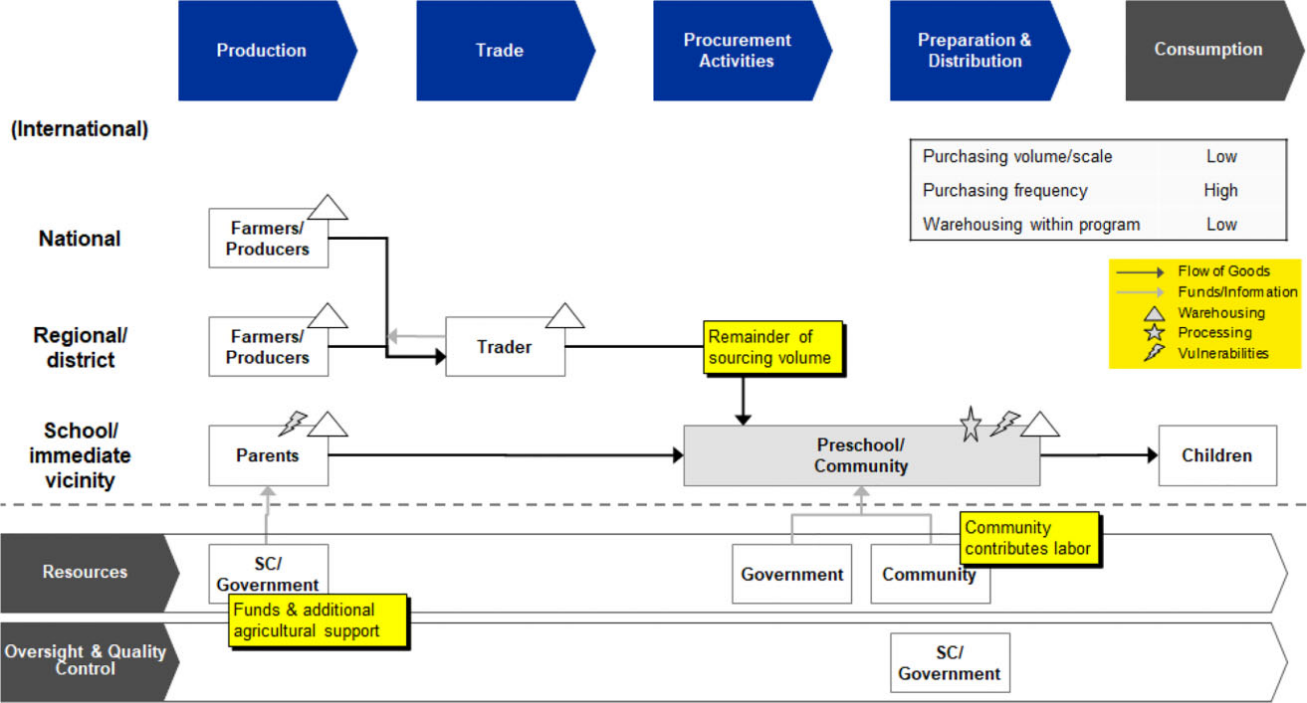
Stylized supply chain for the integrated farm-to-preschool model in Malawi (Source: Authors, adapted from Gelli and Suwa^15^). Vulnerabilities in this model include production risk for parents (smallholders) and food safety risks at preschool level.

The intervention can also be seen as a way of improving the community-based preschool meal service delivery, complementing the preschool meal service with BCC around agriculture and nutrition practices. Conceptually, the program shares common features with HGSF and, in particular, the integrated farm-to-school supply chain. Mapping the program activities to a standardized supply chain model for school meal programs highlights these similarities, with the main difference being that the program targets preschools rather primary schools, as shown in Figure 2 ^15^. The supply chain model shows how the program is linked to agricultural production, driving contributions and sourcing for preschools, where meals are prepared, distributed, and, finally, consumed. Applying this school meals supply chain framework will also allow for comparisons of the preschool meal service delivery with operational benchmarks on unit costs in the literature.

A rigorous impact evaluation of the NEEPIE program was conducted by the International Food Policy Research Institute in 2015 to 2016. The Nutrition Embedding Evaluation Program-Impact Evaluation was a cluster randomized controlled trial (cRCT) designed to examine the effectiveness of using community-based ECD centers as a platform to improve preschool meal delivery by improving caregiver knowledge and nutrition practices, promoting household food production, dietary diversity, and nutrition among preschoolers and their younger siblings.^16^ This evaluation showed that implementing an integrated agriculture and nutrition intervention through an ECD platform benefitted children’s diets and reduced stunting prevalence by 17 percentage points (*P* < .05) among younger siblings of targeted preschoolers in the program.^17^ However, important questions remained on the NEEPIE program costs and, in particular, on costs borne by the community.

## Methods

A cost analysis was conducted from the perspective of the nongovernmental organization (NGO) provider, Save the Children, and the beneficiaries. The analysis estimated the incremental financial and economic costs of implementing an integrated agriculture and nutrition intervention through the existing ECD program targeted to preschool children. Any costs related to external research, such as the program impact evaluation, were excluded from the analysis. The study was guided by two related frameworks. The first framework was developed as part of the Strengthening Economic Evaluation for Multisectoral Nutrition Strategies (SEEMS-Nutrition) initiative.^1^ The SEEMS-Nutrition framework provides a standardized approach for data collection, cost coding, allocation, and integration as well as for the final cost analysis.^18^ The second framework uses a set of standardized activity categories from a school feeding supply chain to facilitate benchmarking and comparisons with other school meal programs.^15^

A mixed-methods approach was used to measure and allocate resource use and costs for program activities and inputs using project-level financial expenditure data combined with micro-costing to allow for estimation of both financial and economic costs. This approach builds on the Activity-based Costing – Ingredients method^19,20^ that uses accounting methods for allocating expenditures by program activities and combines it with the ingredients method that calculates costs for all resources used, including donated or volunteer personnel time and supplies. Financial costs capture actual expenditures for program implementation on an annual basis. Economic costs include the opportunity cost of donated time by community members and other CBCC-level stakeholders involved in preschool meal provision. This method has been applied to assess the total costs of complex nutrition interventions in several settings.^21-24^ We first identified all major program-related activities over the course of the 1-year program and captured the quantities and costs for all inputs associated with start-up and recurrent activities. Start-up activities included the development of agricultural production and training materials, the training of trainers for parenting, agricultural extension and nutrition, support for start-up program activities from Save the Children personnel (coordination, supervision, monitoring, training, materials development), and initial community investments such as CBCC construction. Recurrent activities included community-level trainings on agricultural production, food processing, nutrition, parenting practices, and VSL. Recurrent household-level activities included agricultural training and the provision of agricultural inputs (Table 1). Next, we considered the community-level activities and inputs, describing these in terms of fixed and variable costs. Community-level input categories included fixed costs such as capital equipment, construction materials, and durable kitchen utensils, as well as variable costs for personnel, agriculture production supplies, food provisions, and cooking supplies (Table 2).

**Table 1.** NEEPIE Activities Mapped to SEEMS Standardized Activity Categories.

Activity	Definition
Start-up costs	
Planning/microplanning	Planning, stakeholder, review and close out meetings, program design
Awareness raising/sensitization	Awareness raising and sensitization at all levels (government, regional, community) by extension and community agents, child protection officers
Training	Agricultural production training of trainers (TOT), nutrition TOT, village and savings loans TOT, parenting TOT
Materials development	Manual development for agricultural production training; materials development for nutrition behavior change (BCC) training
Recurrent costs	** **
Management	NGO personnel costs for ongoing project management
Monitoring and evaluation	Designing and implementing program monitoring and evaluation; does not include impact or process evaluation activities of the program
Distribution of inputs	Distribution of agricultural inputs (seeds and vines) and chicks (poultry)
Provision of school meals	Preparing and serving meals, managing food stocks, maintaining kitchens and canteens
Home visits: Agriculture extension	Agricultural extension technical support to households
Establishing and running community groups	Caregiving of children, serving on CBCC committee, maintaining CBCC garden, construction and maintenance of CBCC
Integration and coordination	Integration, monitoring and evaluation, management
Training	Agricultural production training (community level), nutrition and food processing training (community level), village savings and loans trainings (community level), parenting trainings (community level)

Abbreviations: BCC, behavior change communication; CBCC, community-based childcare center; NGO, nongovernmental organization; NEEPIE, Nutrition Embedding Evaluation Program Impact Evaluation; SEEMS, Strengthening Economic Evaluation for Multisectoral Strategies.

**Table 2.** NEEPIE Inputs Mapped to SEEMS Standardized Input Categories.

Input category	Description
Personnel	Paid labor (NGO staff, government workers)
	Volunteer labor (community members)
Supplies	School meal or food preparation inputs (in-kind food contributions, condiments, fuel) Kitchen and canteen construction materials and supplies CBCC construction materials Storage maintenance
Agriculture supplies	Agricultural inputs: Seeds, poultry, fertilizer
Fuel and maintenance	Vehicle fuel, insurance and maintenance
Travel/per diem/allowances	Per diem and travel allowances
Equipment	Purchased materials for meetings
Mixed inputs	Venue, accommodation, meals for trainings
Overhead	CBCC maintenance and repairs

Abbreviations: CBCC, community-based childcare center; NGO, nongovernmental organization; NEEPIE, Nutrition Embedding Evaluation Program Impact Evaluation; SEEMS, Strengthening Economic Evaluation for Multisectoral Strategies.

Lastly, the program activities are aligned to a nutrition-sensitive value (NSV) chain typology of interventions (Table 3). The concept of NSV chains builds upon supply chain models with a focus on smallholders and increasing value along the chain from a nutritional perspective. Nutrition-sensitive value chains provide a framework for mapping and comparing intervention strategies and potential entry points.^25^ For example, the NEEPIE program activities are mapped to the supply chain categories of production, trade, and procurement in Figure 2. These activities are categorized as supply-side activities according to the NSV chain typology in Table 3. Similarly, program activities aligned to preparation, distribution, and consumption are categorized as demand-side activities. Caregiving labor contributed by the community is categorized as part of the enabling environment. This additional step will allow for meaningful future comparisons with other multisectoral nutrition-sensitive interventions using the SEEMS-Nutrition framework approach.

**Table 3.** Nutrition-Sensitive Value Chain Activity Coding.

NSV intervention typology	Entry point	Activity
Increase supply	Diversification/promotion of nutritious crops	Materials development (extension training) Home visits: Extension
Increase demand	Behavior change communication	Materials development (nutrition behavior change communication training) Provision of school meals
Enabling environment	Strengthening childcare and parenting practices	Serving on CBCC committees, Parenting trainings, Parental caregiving at CBCC
Shared program costs allocated to above 3 typologies	Coordination	Planning/microplanning Integration and coordination Monitoring and evaluation, Management

Abbreviation: CBCC, community-based childcare center.

### Data Collection

Financial and economic cost data collection occurred throughout the duration of the NEEPIE study, spanning a multiyear period between September 2015 and October 2019. The intervention began in December 2015 and was implemented for 12 months. Data collection for the parent cRCT began in November 2015 (baseline), midline in April 2016, and endline in November 2016, which included household surveys and in-depth CBCC/community surveys. A second-year follow-up survey was also conducted as part of the cRCT 1 year after the program ended to examine the sustainability of program impacts.

To estimate financial costs, we collected program-level expenditures from Save the Children administrative records for the 12-month program implementation. These expenditures were then coded using predefined cost categories. To compliment the expenditure records, we conducted in-depth CBCC/community surveys periodically between October 2015 and 2017 to obtain detailed information on the quantities of resources used to implement the integrated agriculture and nutrition package. These resources included staff time allocation, infrastructure investments, meal provision, and CBCC collective gardens where food for the program was grown (see study protocol for details^16^). Price data for the food items used in the meals were collected using standard market-level surveys in the 4 main markets in the study area. There were 60 total CBCCs included in the NEEPIE intervention, 30 of which were treatment CBCCs and 30 of which were control CBCCs. The 60 CBCCs served 1248 preschool children (36-72 months) and 304 younger siblings (aged 6-24 months).

As the study captured incremental costs to the base ECD program, costs were not collected at control CBCCs. We used an in-depth survey to collect retrospective data from all 30 treatment CBCCs covered using semistructured questionnaires. The questionnaires were designed to capture the opportunity costs of beneficiaries (labor and out-of-pocket expenditures) to feed into the overall cost analysis. The CBCC-level questionnaire covered both cash and in-kind contributions for agriculture production and complementary nutrition education to improve consumption of nutritious foods. As per the design of the intervention, community engagement and contributions were critical to successful implementation. Households in intervention villages contributed volunteer time to CBCCs as well as in-kind donations. Volunteer labor was contributed by women from participating households in preparing school meals, tending gardens, caring for children during CBCC hours, and participating on CBCC management committees. Men from intervention households generally provided support to construct or maintain CBCC buildings, and some participated in CBCC committees. Donations for CBCC meals were made in several forms, either as foodstuffs produced at the household level or as food purchased by small household cash contributions or pooled community contributions. Despite the use of a semistructured questionnaire to gather data on costs, it was not possible to obtain accurate day-to-day expenditures at the CBCC level due to the low level of record-keeping. As such, we generated estimates of the total incremental cost of implementation considering all activities required to provide the intervention service, using data collected from the different CBCCs.

The economic costs obtained from the survey on community contributions or donations included:

- Cash value in Malawian kwacha (MKW), such as cost of pots, utensils, and stoves for the CBCC
- In-kind costs (MKW), such as in-kind foodstuffs contributed to the program
- Volunteer time costs (hours per day), for example, time spent caregiving for children at the CBCC or in the construction and maintenance of CBCC buildings

At the household level, data on opportunity costs were collected using a time allocation module in the household survey from the external program evaluation. This time allocation module was based on the standard, validated module developed for the Women’s Empowerment in Agriculture Index^26^ and was adapted to include NEEPIE program-specific activity categories.

### Data Analysis

The overall analytical approach was to integrate total economic costs from the CBCCs and households—in voluntary labor and other contributions—with the NGO expenditure data to arrive at total incremental costs of the program. Expenditure data included the salaries of program staff, frontline workers who provided extension and nutrition support, and government workers as well as NGO costs for agricultural inputs, training, and other supplies including vehicle use and fuel. Program staff were interviewed retrospectively to estimate the allocation of their salaried time to program activities during the implementation period.

The next step was to incorporate the opportunity cost of volunteer time donated to program implementation. The opportunity cost of time invested in the program was calculated by multiplying the hours of voluntary beneficiary labor per child per year donated to the program by 50% of the minimum wage for a 19-year-old apprentice unskilled worker in Malawi, which is US$23 per month, as it has been reported that actual wages in Malawi are less than half of legal minimum wages.^27^ This wage rate was used to reflect the labor profile of the equivalent type of worker. Apprentice wages were used as the participating households are poor agricultural households with low levels of education (55.1% with a primary education and only 16.3% possessing any secondary education); thus, the average wage representing formal employment is not representative of this population. For this reason, we utilized an apprentice wage as it was more representative; however, we also modeled other wage scenarios. We conduct sensitivity analyses to model different wage rate scenarios (50% apprentice wage, 50% minimum wage, and 50% average wage) to provide a cost range of the lower and upper bounds (Table 7). The total time in hours donated in the program by volunteers was recorded as part of the CBCC data collection activities described above as well as through the time allocation module in the household survey.^28^ The CBCC survey collected volunteer contributions from both men and women for specific CBCC activities (construction, maintenance and repairs, committee participation, meal preparation) as captured in the CBCC survey. These CBCC costs also captured start-up investments of labor such as preschool construction. The time allocation module from the household survey measured female caregiver beneficiary time over a 24-hour period during intervention activities. There was a statistically significant difference (*P* < .05) between the treatment and control groups in the number of minutes devoted to caregiving and other program activities by beneficiaries as compared to the control group. This additional time was valued at the same wage rate as above (50% minimum wage for unskilled apprentice). Beneficiary time contributions were allocated as 50% to the activity of establishing and running community groups and 50% to the provision of school meals.

To estimate the annual cost of the program per child, we aggregated estimates of individual cost items per child per day based on data collected from each CBCC to arrive at an average daily cost per child per day and ultimately an annual cost per child. The following steps were used to estimate the annual cost per child enrolled in the CBCC.

First, using resource and cost data collected from each participating CBCC, we obtained a daily program cost for each activity. For daily CBCC food costs, we estimated the quantity of each of the food items provided and then multiplied each item by the average price of that item obtained from the local market price survey. A summary of cost calculations can be found in Table 4. Daily food costs were added to other recurrent costs and allocated to the school meals provision activity category as indicated in Table 5. Second, after obtaining the daily CBCC program cost per item, we estimated the daily cost per child per item. We divided the daily CBCC program cost per item by the number of children per CBCC to arrive at the daily cost per item per child for each CBCC. Third, we estimated an average cost per item per child per day across all 30 CBCCs in the program. Fourth, we obtained an average annual cost per child for all program costs. Thus, we summed all the average program costs per item per child per day to obtain an average total program cost per child per day. This cost was multiplied by 200 days to obtain an annual cost per child, assuming a 200-day school year as per the Malawi school calendar.

**Table 4.** Cost Calculations Summary.

Step	Calculation
1. Obtain a daily cost for each program input or activity per CBCC.	
2. Obtain a daily cost per child for each program cost item per CBCC.	Divide the cost value by the number of children in the CBCC.
3. Obtain an average daily cost per child for each program cost item across CBCC.	Calculate a mean value of the daily per child cost for each item across CBCCs that have reported that cost item.
4. Obtain an average annual program cost per child across all CBCCs	Sum the average daily per child costs for each program cost to arrive at an estimated total program cost per child. Then multiply this by 200 days to obtain a total annual program cost per child.
5. Calculate the opportunity costs of volunteer time (hours per child per year) donated to the NEEPIE program	Multiply the hours per child per year invested in the program by 50% of US$23 per month, half the minimum wage for apprentice (unskilled) worker in Malawi.

Abbreviation: CBCC, community-based childcare center.

**Table 5.** Summary of Costs by Activity for the Integrated Agriculture and Nutrition Intervention in Malawi.

	USD	Percent
Input		
Personnel	$75770.07	38.4
*Hired*	*$60,493.18*	*30.7*
*Volunteer*	*$15,276.89*	*7.7*
Equipment (capital goods, including vehicles)	$1301.31	0.7
Supplies (donated)	$37152.14	18.8
Agriculture supplies	$3585.16	1.8
Fuel and maintenance	$1219.05	0.6
Travel/per diem/allowances	$44356.00	22.5
Mixed inputs	$32903.80	16.7
Overhead	$1089.84	0.6
Total	$197377.37	100.0
Stage		
Start-up	$46749.33	23.7
Recurrent	$150628.04	76.3
Total	$197377.37	100.0
Activity		
Materials development	$5801.97	2.9
Training	$89911.96	45.6
Distribution of inputs	$3585.16	1.8
Integration and coordination	$1433.33	0.7
Provision of school meals	$36724.42	18.6
*Food (donated)*	* $32423.10*	*16.4*
*Supplies (donated)*	*$1653.86*	*0.8*
*Volunteer time (meals)*	* $2540.35*	*1.3*
*Other*	* $107.11*	*0.1*
Establishing and running community groups *(volunteer)*	$12736.54	6.5
*Volunteer time (construction, running groups)*	*$9661.37*	*4.9*
*Supplies (donated)*	*$3075.18*	*1.6*
Home visits: Agriculture extension	$10241.56	5.2
Monitoring and evaluation	$24308.62	12.3
Planning/microplanning	$4327.64	2.2
Awareness raising/sensitization	$743.94	0.4
Management	$7562.24	3.8
Total	$197377.38	100.0

Fifth, in order to understand costs by a set of standard activity codes, we retroactively mapped the NEEPIE activity and input costs to the standardized SEEMS-Nutrition cost categories. Table 1 illustrates how the NEEPIE costs are applied to the SEEMS-Nutrition activity categories. Table 2 allocates the NEEPIE input costs to the SEEMS-Nutrition input cost categories. For example, the program activity of agricultural extension manual development was coded as the SEEMS standard activity cost category of materials development. Then, an SEEMS standard input cost category was assigned to each activity. In the case of materials development, the assigned input was personnel, as the cost associated with the activity of materials development was staff time to develop the manuals.

Lastly, Table 3 presents the cost data categories aligned with the NSV chain model. Intervention activities were coded to an NSV chain typology, using a similar mapping exercise as with the SEEMS activity coding, but framed within 3 intervention typologies related to increasing demand, increasing supply, and the enabling environment. Because there are some activities such as coordination activities and integration meetings with partners that extend across both agriculture and the education sectors, we allocated these shared program costs in proportion to the share of the intervention typologies’ cost out of the total program costs (ie, increasing demand activity costs divided by the total program costs, etc). An example of how activities were coded to the NSV intervention typology is provided in Table 3.

Capital costs were annuitized over the period of program implementation using a discount rate of 3% following World Bank recommendations. Annuitization enables an equivalent annual cost to be estimated and reflects the value in-use of capital items, rather than reflecting when the item was purchased.^29^ The assumed useful life of all relevant school-level capital equipment was set to 10 years (except for the development of the agricultural production manual for the program, which was set to have a useful life of 5 years). All costs were incurred in MKW and converted to USD using official exchange rates, based on an average yearly exchange rate of US$1 to 714 MKW in August 2017. Cost data were coded and analyzed using Microsoft Excel. Time allocation data from the survey were analyzed in Stata version 14.2.

### Sensitivity Analysis

To obtain an estimate of how costs of the program could vary, a range of costs were calculated based on different scenarios, including variations of wage, training program intensity, and beneficiaries:

- Wage variations: The opportunity cost of the program in USD was calculated by obtaining an approximate hourly value of the unskilled labor wage, the legal minimum wage, and the average wage. These were then halved since it has been reported that actual wages in Malawi tend to be less than 50% of legal wages.^27^ Each of these 3 wage estimates was multiplied by the total number of hours invested in the program by volunteers in the community, resulting in varying estimates for the monetary opportunity cost of time invested in the program by volunteers.
- Training variations: The cost of training communities to run the program was found to comprise a significant proportion of total program costs (46%). Trainings were run during the first and only year of the program. Since the magnitude of follow-up training required over a hypothetical 5-year program life span could vary, 3 different scenarios were considered: In the first scenario, the training was run in the first year only, with no follow-up training in the subsequent 4 years of the program life span. In the second scenario, training was run in the first year combined with yearly follow-up training that was less intensive, assumed at 75% of the first-year training in each of the subsequent 4 years of the program life span. In the third scenario, the training program was assumed to be run at the same initial yearly cost for all 5 years of the program’s duration.
- Beneficiary variation: The total cost of the program (including cash, in-kind, and opportunity costs) was initially reported as a cost per year per child enrolled in the participating CBCCs. Since other members of the household have also been found to benefit from the program (eg, the nutritional information imparted to communities by the program enhances the nutritional quality of their meals as found in the impact study), a total program cost per beneficiary was also calculated by dividing the cost per child by the average number of members per household in the communities participating in the study.

### Cost-Efficiency Analysis of the Preschool Meal Service Delivery

Process and output data covering the adequacy of the meal service delivery (eg, meal quality) were collected from CBCC and household-level surveys using standardized data collection forms. Food composition data were retrieved from a Malawi-adapted food composition table^30^ and used for a linear modeling analysis of the CBCC menus for meals provided the day before the survey. Output data were combined with costs to provide estimates of unit costs or cost-efficiency, including costs per preschool child served, cost per kilocalories delivered, cost per mg iron delivered, and cost per 100 mcg vitamin A delivered. To compare with operational benchmarks for school feeding in the literature, the total cost per child served per year was standardized to a 200-feeding-day year and 700 daily planned kcal.^31^

## Results

### The Costs of the Integrated Intervention

Data were collected from 30 CBCCs that received the integrated intervention and were operational during the survey period. The results of the cost analysis are summarized in Table 5. Financial costs of the program totaled US$147 916, and total economic costs were US$197 377. The total incremental cost per child per year of the program was US$160. Table 6 summarizes the main cost drivers of the program by activity type, which were primarily training (46% of total costs), school meals (19%), monitoring and evaluation (12%), and establishing and running community groups (6%). Nearly a quarter of total costs (24%) were start-up costs and the rest were recurrent costs (76%). The main costs by input category included personnel costs (38% of total), travel/allowances (23%), supplies (19%), and mixed inputs (17%).

**Table 6.** Cost Drivers for the Integrated Agriculture and Nutrition Intervention in Malawi (Source: Authors).

Activity category	Percentage of total cost
Training	46
School meals	19
Monitoring and evaluation	12
Establishing and running community groups	6
Home visits: Agriculture extension	5
Management	4
Materials development	3
Planning/microplanning	2
Distribution of inputs	2
Integration and coordination	1
Awareness raising and sensitization	<1

Community contributions ($49460.97) accounted for US$40.08 per child per year, or 25% of total program costs, driven by food contributions (16% of total program costs and 66% of total community contributions) and volunteer time in caregiving, meal preparation, and other support (accounting for 7.7% of total program costs and 25% of total community contributions).

We also examine variability by CBCC size to show variation of CBCC costs. The mean cost per CBCC is $3360, with the lower ($1920) and upper bounds ($7360). The cost per CBCC is generated by taking the upper and lower bounds for CBCC size by enrolment and multiplying by the cost per child.

**Figure 3.**
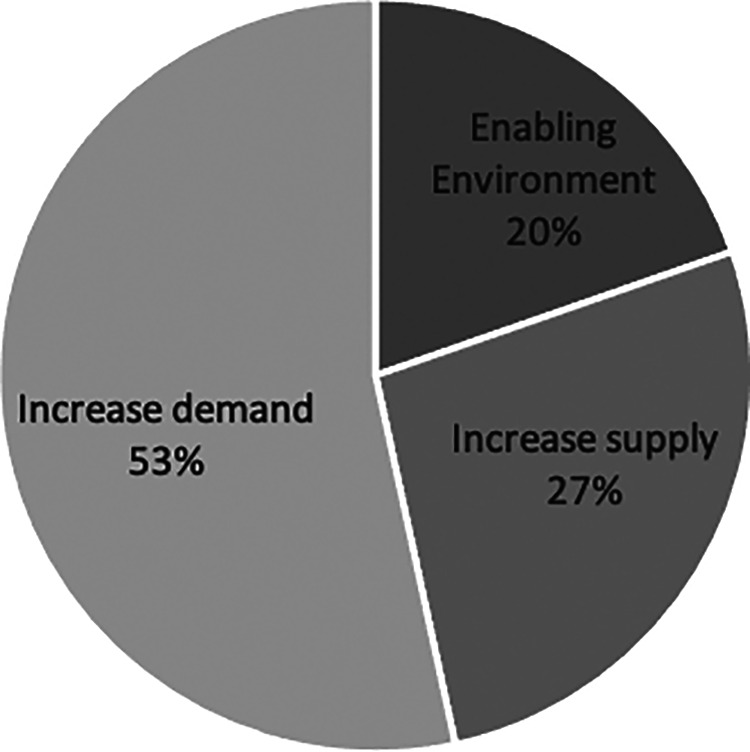
Cost drivers mapped to the nutrition-sensitive value chain typology for the integrated agriculture and nutrition intervention in Malawi (Source: Authors).

When exploring how program activities map to the NSV chain typology (Figure 3), approximately half the costs were incurred by demand-side intervention components. These included activities oriented to increasing demand for nutritious food, for example, activities related to BCC and the actual provision of school meals. Demand-side activities occupied close to double the share incurred by supply-side intervention components such as agricultural extension home visits, which are related to diversification or the promotion of nutritious food production. Twenty percent of costs were associated with activities related to promoting an enabling environment for the program, such as ECD-supporting activities like strengthening childcare and parenting practices. Shared activities such as coordination costs were allocated proportionally across all 3 NSV categories. Greater detail on the activities composing each of the NSV categories can be found in Table 3.

### Sensitivity Analysis and Comparisons With Meal Service Delivery Benchmarks

The evidence from the 3 alternative sensitivity analysis scenarios using varying wage rates (Table 7) suggested that the costs per beneficiary per year could range from a maximum of US$172 (in the case of repeated intensity trainings with wages equivalent to 50% of average wage) to a minimum of US$41 (when considering total household beneficiaries).

**Table 7.** Summary of Sensitivity Analysis Scenarios.

Varying wage rates scenario	50% of apprentice wage	50% of minimum wage	50% of average wage
Total yearly program cost (USD) per preschool child	156	157	172
Varying training intensity scenario	Training in year 1 only	Training repeated at 75% intensity in years 2-5	Training repeated fully each year
Total yearly program cost (USD) per preschool child	63	135	158
Varying beneficiary scenario	Cost per preschool child	Cost per beneficiary	
Total yearly program cost (USD)	160	41	

The findings on the cost-efficiency metrics are summarized in Table 8. Interestingly, there were no benchmarks for the farm-to-school/preschool model in the literature and comparisons were only available with a centralized operational model, or a cluster kitchen model. The NEEPIE model, cost standardized using the parameters from the benchmarks of school meal programs, was US$254 per preschool child, US$36 per 100 kcal, US$32 per mg of iron delivered, and US$308 per 100 mcg of vitamin A delivered (Table 8). The cost-efficiency of vitamin A delivery will require further investigation as the vitamin A content of meals delivered through the integrated agriculture and nutrition intervention is underestimated in this analysis. Although the school meals contained both orange maize and orange-fleshed sweet potato (OFSP)—foods rich in vitamin A—we did not account for the nutrient content estimates of these meals.

**Table 8.** Summary of Cost-Efficiency Metrics for the Integrated Farm-to-Preschool Model in Malawi and Comparisons With School Feeding Benchmarks and Other Implementation Models Using the Same Costing Methodology.^a^

Model	Standardized cost per child^b^	Cost per 100 kcal	Cost per mg of iron	Cost per 100 mcg of vitamin A
NEEPIE Malawi farm-to-preschool model	254	36	32	308
Operational benchmark from centralized model^31^	45	12	10	21
Cluster kitchen model^15^	117	18	33	38

Abbreviation: NEEPIE, Nutrition Embedding Evaluation Program Impact Evaluation.

^a^ All costs in USD and adjusted for inflation in 2017.

^b^ Cost standardized to 700 kcal. No benchmarks exist for farm-to-school/preschool model.

## Discussion

Nutrition-sensitive approaches, such as school feeding and ECD programs, have the potential to provide platforms to scale up nutrition interventions. However, despite the recent progress in generating evidence on the effectiveness of these programs, there are important gaps on the costs of implementation. Moreover, despite the popularity of school meal programs, there is little rigorous evidence on the total incremental costs of program implementation.^12^ This gap is particularly important when examining implementation models that require contributions at the community level, where opportunity costs like volunteer time in food preparation and child care, or in providing in-kind food donations, are often overlooked.^13^

The integrated agriculture and nutrition intervention implemented in Malawi, an example of an integrated farm-to-preschool school feeding model, incorporates a range of innovations in program design. The intervention used preschool meals as a platform to implement a range of agriculture and nutrition activities aimed at improving production and consumption of nutritious foods. Unlike traditional school meal programs, no food or cash transfers from external agencies or government were involved. Similar to the integrated farm-to-school model that has been implemented in Cote d’Ivoire, for example, it strives to link school meals to in-kind donations of nutritious foods from the community.^15^ However, unlike the traditional farm-to-school model where donations are encouraged but the meals rely on government food and cash transfers, the meal provision in the NEEPIE model relies entirely on voluntary contributions from parents and other community members. As a result, community contributions accounted for 25% of total costs, equivalent to approximately US$40 per child per year. These figures are remarkable, considering that the intervention was scaled up in high poverty rural areas with chronic food insecurity. The only other study that collected data on community contributions found that these contributions ranged from 0% in Lesotho to 15% in Kenya.^13^ In the Malawi case, food donations accounted for 66% of total community contributions, which does not include the additional volunteer time in caregiving, meal preparation, and other support. The financing of the model implemented in Malawi is thus heavily subsidized by communities receiving the program, highlighting an opportunity for scale-up as part of the ECD policy of the Government of Malawi’s (GoM).^32^

Subsequently, the GoM recently partnered with the World Bank to scale up this intervention model nationally under its “Investing in Early Years for Growth and Productivity in Malawi” project. This approach is important as the program targets vulnerable children in the age gap between the first 1000 days and traditional school age. The SEEMS-Nutrition consortium is currently working with the GoM to collect and analyze the costs of the government-implemented intervention.

Our analysis highlights the level of contribution by communities, which was the intention of the program’s design and also a positive indicator of the investment of households in the program. However, the intensity of community engagement could also be a potential limiting factor affecting feasibility and generalizability. The high level of community engagement might not be found in all settings, particularly in urban contexts where communities may be less cohesive. Second, although participants may reprioritize or change their daily tasks while contributing to program activities, it is critical that they do not become overburdened. A separate study examined whether female participants were burdened by NEEPIE activities and found that although there was a modest increase in daily caregiving, they did not consider contributions burdensome or unmanageable.^28^ Regardless, these results provide a strong case for conducting economic evaluations to identify and acknowledge the significant costs of participation to communities. An economic evaluation is underway by the SEEMS-Nutrition consortium to conduct further cost–benefit analyses of the NEEPIE program.

The intervention also benefitted other household members alongside the participating preschoolers receiving meals, including improved caregiver knowledge of nutrition, diet diversity and linear growth in their younger siblings, and production of nutritious foods. These additional benefits suggest that the estimates of cost per beneficiary, rather than cost per child receiving meals, may provide a more accurate metric for this particular type of intervention, thus increasing considerably its cost-efficiency.

We still have much to learn by exploring the costs of multisectoral agriculture-nutrition programs. Examining costs with the SEEMS-Nutrition activity and input categories and using the NSV chain typology expands our ability to disaggregate costs and to analyze resource allocation patterns. Exploring cost components or cost shares by the SEEMS-Nutrition activity, input, and value chain intervention typology categories has the potential to enhance future cost comparability with other multisectoral interventions to improve nutrition in cases where the methods for measuring and valuing resources and costs are transparent (ie, clearly explained).

There is scant evidence of agricultural value chain interventions improving nutrition^33^, making comparisons difficult, particularly as many of these interventions are not designed to address nutritional outcomes. Comparisons across studies are also challenging due to the different methodologies involved, including on how to account for coordination costs. For example, in this study, costs associated with activities for the coordination across sectors accounted for approximately 18% of total expenditures. How these costs compare to other models of implementation remains an important question for program and policy stakeholders looking to scale up these types of programs. The Global Health Cost Consortium (GHCC) reference case on global health costing notes that resources that occur above the delivery site, whether that is the clinic or community, are often excluded. The GHCC recommends that these costs be considered in the same way as on-site costs, rather than arbitrarily omitted. When these costs are difficult to capture, the omission should be clearly stated, and any bias reported.^34^ A study of an integrated agriculture, health, and nutrition project implemented in Kenya estimated above-site integration and coordination activities as monthly feedback meetings and any other coordination or monitoring activities that included participation by all partners and implementing agents. They found that these above-site costs accounted for 27% of total incremental costs.^24^

For those value chain interventions that do have nutrition objectives, outcomes have been mixed. For example, one dairy-focused NSV chain intervention, similarly targeted to preschoolers, raised hemoglobin but did not reduce anemia.^35^ In programs with a deliberately stated nutrition objective such as NEEPIE, using an NSV chain framework to map costs allows for a better understanding of how program activities are aligned to value chain typologies (ie, if program activities are more focused on driving demand in the beneficiary population vs increasing the supply of nutritious foods). This analysis could aid program implementers in decision-making around future program design. For example, if a program did not have intended impacts on driving consumption and, through an examination of costs, it emerged that most costs were directed toward supply-side activities, this might suggest more resources to be directed to demand-side activities such as BCC for the consumption of nutritious foods or to child feeding practices. This mapping also could aid future comparisons with other complex, multisectoral NSV interventions, to better chart the pathways through which impact is occurring in each program and as well as to trace the flow of program resources to those activities linked with the pathways. This approach also shows relative trade-offs in how to allocate resources when investing in different NSV chain approaches.

Likewise, the mapping of costs to program activities highlights the drivers of cost. This is helpful for implementers—who are interested in controlling program costs and increasing efficiencies—to understand which activities comprise the majority of costs and allowing for identification of opportunities for economies of scale. For example, travel costs/per diems comprise 22% of input costs and 45% of activity costs are related to training. The program could investigate whether travel could be made more efficient for actors delivering trainings such as delivering trainings at regional hubs. Of course, such adjustments must assess potential costs to program participants, such as transferring travel costs onto beneficiaries. Policymakers also benefit from understanding cost drivers, as with the ECD GoM scale-up, to understand how program costs change over time, to pick and choose program activities for policy design, or to identify synergistic activities with extant programs (ie, taking advantage of frontline worker visits for other programs).

Comparisons with other integrated agriculture and nutrition interventions are not straightforward due to the heterogeneity in intervention design and absence of a standardized costing approach that captures activities across sectors. However, under the current scenario, the cost per beneficiary and cost per household reached are within the same range of the few estimates found in the literature. The costs of an integrated agriculture and health intervention promoting OFSP production and consumption in Western Kenya (Mama SASHA) were estimated at US$155 per pregnant women reached or US$110 per beneficiary if both pregnant mothers and infant children were included.^24^ Another study estimated the costs over 10 years of delivering an enhanced homestead food production intervention in rural Cambodia to be US$929 per household.^36^ In Zimbabwe, a study found that the average costs per household reached of an intervention providing community vegetable gardens for people living with HIV were US$1890 in 2010.^37^ A cross-country study in Ethiopia, Nigeria, and India found that the costs per child reached of agriculture and nutrition intervention varied widely, ranging from US$2650 for a livestock transfer program to US$0.58 for a media and education campaign.^38^ In comparisons with school feeding programs, the evidence suggests that integrated farm-to-preschool model as implemented in Malawi is generally less cost-efficient than other school feeding program models (except for iron; Table 8) if one considers only the preschoolers receiving meals as the beneficiaries of the program. As the NEEPIE program provided a range of services and benefits beyond the school meals that reached other beneficiaries alongside preschoolers, comparisons with school feeding benchmarks may not be entirely relevant.

The approach under development through the SEEMS-Nutrition framework aims to address this challenge of comparability through standardization of methods, tools, and analysis. Future work will outline this approach as well as apply it to other multisectoral programs and contexts, building a toolbox for economic evaluation of multisectoral nutrition programs as well as growing the evidence base.

### Limitations

The study findings are limited by several important considerations. First, it was not possible to obtain an accurate picture of the day-to-day expenditures at the CBCC level due to the low level of record-keeping. As such, in our analysis, we used the existing data from the different CBCCs to generate estimates of the total incremental cost of implementation considering all activities required to provide the intervention service. The data used for the estimations at community level were based on caregiver recall, which is likely to have measurement error. However, the per-child estimates of the different community-level costs compare relatively well with those from similar studies in similar contexts.^13,15^ Another important consideration involves the opportunity cost of time invested in the program by community volunteers; when this is considered in USD, it appears as a relatively low cost due to the low wages for unskilled labor in Malawi. In actual hours worked, this translates to approximately 60 h/child/yr, which perhaps provides a better reflection of the opportunity cost of the intervention. As a function of caregivers’ total available time, this represented only a small percentage. However, the intervention is intensive and involves a considerable amount of inputs. In the absence of cost-effectiveness analysis, an important gap remains in terms of understanding the value of scaling up these types of integrated interventions. However, because of positive impacts with large effect sizes on preschoolers, younger siblings, and households and how the program holds up to other benchmarks, it is suggestive that the program provides significant benefits for the cost invested. Additionally, as the benefits accrue to different groups, the cost–benefit is not straightforward and provides a challenge for economic evaluation, which will be presented in a future paper.

### Conclusion

By assessing the total economic costs of implementing the community-based preschool meal program, this study is the first to our knowledge to provide evidence on the total incremental costs of implementing an integrated agriculture-nutrition intervention through an ECD platform. Cost per beneficiary estimates compare favorably with similar interventions. Further research is needed that applies a standardized economic evaluation framework to these types of multisectoral interventions.

## Note

1 The SEEMS project is funded by the Bill and Melinda Gates Foundation and is led by the University of Washington in collaboration with the International Food Policy Research Institute.
